# Blockade of Extracellular ATP Effect by Oxidized ATP Effectively Mitigated Induced Mouse Experimental Autoimmune Uveitis (EAU)

**DOI:** 10.1371/journal.pone.0155953

**Published:** 2016-05-19

**Authors:** Ronglan Zhao, Dongchun Liang, Deming Sun

**Affiliations:** 1 Doheny Eye Institute and Department of Ophthalmology, David Geffen School of Medicine at UCLA, Los Angeles, CA, 90033, United States of America; 2 Department of Medical Laboratory, Key Laboratory of Clinical Laboratory Diagnostics in the University of Shandong, Weifang Medical University, Weifang, 261053, Shandong, China; Oregon Health & Science University, UNITED STATES

## Abstract

Various pathological conditions are accompanied by ATP release from the intracellular to the extracellular compartment. Extracellular ATP (eATP) functions as a signaling molecule by activating purinergic P2 purine receptors. The key P2 receptor involved in inflammation was identified as P2X7R. Recent studies have shown that P2X7R signaling is required to trigger the Th1/Th17 immune response, and oxidized ATP (oxATP) effectively blocks P2X7R activation. In this study we investigated the effect of oxATP on mouse experimental autoimmune uveitis (EAU). Our results demonstrated that induced EAU in B6 mice was almost completely abolished by the administration of small doses of oxATP, and the Th17 response, but not the Th1 response, was significantly weakened in the treated mice. Mechanistic studies showed that the therapeutic effects involve the functional change of a number of immune cells, including dendritic cells (DCs), T cells, and regulatory T cells. OxATP not only directly inhibits the T cell response; it also suppresses T cell activation by altering the function of DCs and Foxp3^+^ T cell. Our results demonstrated that inhibition of P2X7R activation effectively exempts excessive autoimmune inflammation, which may indicate a possible therapeutic use in the treatment of autoimmune diseases.

## Introduction

During the past two decades, increasing evidence has shown that tissue stress or damage is closely associated with increased release of ATP from the intracellular into the extracellular compartment; this increased ATP release, in turn, exerts a strong modulatory effect on immune responses and inflammation [1–8]. Many cell types are able to release ATP [3,5,9,10]. Receptors that specifically bind ATP, designated as P2 receptors, are widely expressed on virtually all cell types, including immune cells [11–15]. Activation of P2 receptors by ATP effectively modulates various immune responses. For example, ATP release resulted in a lowered threshold for activation and T cell–mediated immunopathology [3,5,16]; ATP served as a signal amplification mechanism for antigen recognition [2,3,12] and as a costimulatory factor for T cell activation [6,17]. Studies revealed that modulations of the extracellular ATP/adenosine metabolism or manipulation of the binding of ATP metabolites to specific receptors could generate a strong effect on immune responses [6,16,18]. In several animal models studying inflammation and autoimmune diseases, ATP promoted inflammation [19–26] and promoted survival of grafted organs [27,28]. Blockade of ATP binding prohibited the development of diseases such as diabetes and experimental autoimmune encephalomyelitis (EAE) [3,29]. Pharmacological approaches that target eATP signaling are a promising therapy for the treatment of cancer and uncontrolled infections [14,20]. Among the receptors that bind ATP, P2X7R is most abundantly expressed on mouse CD4^+^ T cells [28,30].

Our studies on the effect of extracellular adenosine on autoimmune responses [31–35] have shown that such molecules have a strong effect on this autoimmune response [31,33–36]. Given that adenosine is a metabolite of ATP and that dysfunction of P2X7R signaling impaired T-cell function and suppressed T-cell activation [5,17,37], we wished to determine the effect of extracellular ATP signaling on autoimmune uveitis and the effect of blocking ATP binding on Th1 and Th17 autoimmune responses.

oxATP is a small Schiff-base molecule that irreversibly antagonizes P2X7R activation by eATP [38,39] and that has been found to be the most effective P2X7R inhibitor [40,41]. In this study we show that in vivo administration of oxATP effectively ameliorated induced experimental autoimmune uveitis (EAU) in B6 mice. Mechanistic studies showed that the blocking effect essentially impeded activation of Th17 autoimmune responses. Dendritic cells (DCs) and Foxp3^+^ T cells are crucially involved as major targets of the treatment.

## Materials and Methods

### Animals and Reagents

Female C57BL/6 (B6) mice were purchased from Jackson Laboratory (Bar Harbor, ME); 12- to 16-week-old mice were used in all studies. All mice were housed and maintained in the animal facilities of the University of California Los Angeles. Institutional approval (Protocol number: ARC#2014-029-03A) was obtained from the Institutional Animal Care and Use Committee of the Doheny Eye Institute, University of California Los Angeles, and institutional guidelines regarding animal experimentation were followed. Veterinary care was provided by IACUC faculty. Immunized animal that displays swelling joints were either be humanely euthanatized or administered an analgesic (buprenorphine, 0.1 mg/kg sc. twice daily or ketoprofen, 2 mg/kg sc. daily) until the swelling resolves. By the end of the study, mice were euthanized by cervical dislocation after an injection of over dosed Ketamine and xylazine prior to tissue collection.

Recombinant murine IL-12 and IL-23 were purchased from R & D Systems (Minneapolis, MN). Fluorescein isothiocyanate (FITC)- or phycoerythrin (PE)- or allophycocyanin (APC)-conjugated antibodies against the mouse αβ TCR, IFN-γ, IL-17, Foxp3 and isotype control antibodies were purchased from e-Bioscience (San Diego, CA). ATP and oxATP were purchased from Sigma-Aldrich (St. Louis, MO).

### Induction and Evaluation of EAU

EAU was induced in B6 mice by subcutaneous injection at 6 spots at the tail base and on the flank with an emulsion containing 200 μg of the human interphotoreceptor retinoid-binding protein (IRBP) peptide (IRBP_1-20_) (Sigma-Aldrich) in phosphate-buffered saline (PBS) and complete Freund’s adjuvant (CFA) (Difco, Detroit, MI) and intraperitoneal (i.p.) injection of 300 ng of pertussis toxin. The mice were then randomly grouped and injected i.p. with oxATP in PBS (300 μg/mouse, twice a week) or with PBS alone (vehicle), starting 1 day post-immunization. Mice were examined for clinical signs of EAU three times a week until the end of the experiment (day 30 post-immunization) by indirect fundoscopy, in which the pupils were dilated using 1.25% phenylephrine hydrochloride ophthalmic solutions and 0.5% tropicamide. The fundoscopic grading of disease was performed using the scoring system reported previously [29]. Histopathological evaluation was performed on eye sections on day 21 post-immunization, because the actively induced EAU in mouse is a monophasic disease that peaks at 20–25 days post-immunization. For details of related tissue fixation, embedding and slicing, see previous report [38].

### T Cell Preparation

All αβ T cells used were purified from the spleen or draining lymph nodes of IRBP_1-20_ immunized mice at day 13 post-immunization using an auto-MACS separator system, as described previously [29]. The purity of the purified cells was >95%, as determined by flow cytometric analysis using PE-conjugated antibodies against αβ T cells. The cells were then cultured in RPMI 1640 medium containing 10% fetal calf serum (Corning).

### Generation of Bone Marrow Dendritic Cells

Bone marrow dendritic cells (BMDCs) were generated by incubation of bone marrow cells for 5 days in the presence of 10 ng/ml of recombinant murine GM-CSF and IL-4 (R&D Systems), as described previously [42]. In this study, the BMDCs were generated and cultured with or without oxATP (80 μM). Cytokine (IL-1, IL-6, L-12 and IL-23) levels in the culture medium were measured by ELISA. To determine antigen-presenting function, BMDCs were incubated in a 24-well plate with responder T cells isolated from immunized B6 mice under Th1- or Th17-polarizing conditions. Forty-eight hours after stimulation, IFN-γ and IL-17 in the culture medium were measured by ELISA. The percentage of IFN-γ^+^ and IL-17^+^ T cells among the responder T cells was determined by intracellular staining after 5 days of culture as described above.

### Measurement of Th1 and Th17 Responses

αβ T cells (1.8 x 10^6^) were collected from IRBP_1-20_-immunized B6 mice, with or without oxATP treatment, on day 13 post-immunization. To obtain a sufficient number of cells, we routinely pool the cells obtained from all six mice in the same group, before the T cells are further enriched. The cells were co-cultured for 48 h with irradiated spleen cells (1.5 x 10^6^/well) as antigen presenting cells (APCs) and IRBP_1-20_ (10 μg/ml) in a 24-well plate under either Th1 (culture medium supplemented with 10 ng/ml of IL-12) or Th17 polarized conditions (culture medium supplemented with 10 ng/ml of IL-23) [34,43]. Cytokine (IFN-γ and IL-17) levels in the serum and 48 h of culture supernatants were measured by ELISA (R & D Systems). The percentage of IFN-γ^+^ and IL-17^+^ T cells among the responder T cells was determined by intracellular staining 5 days post in vitro stimulation, and followed by FACS analysis, as described previously [34].

For adoptive transfer, 2 x 10^6^ IRBP-specific T cells isolated from IRBP_1-20_ immunized B6 mice (injected with either oxATP or PBS) on day 13 post-immunization were injected i.p into recipient mice as described previously [44].

### Assessment of the oxATP Effect on DCs

Responder T cells and APCs were separated from immunized B6 mice 13 days post immunization. T cells and/or APCs were pre-incubated with 80 μM oxATP. The T cells and APCs were washed and then co-incubated in the presence of the immunizing peptide IRBP_1-20_ in a 24-well plate, under Th1- or Th17-polarizing conditions. Forty-eight hours later, Cytokine (IFN-γ and IL-17) levels in the culture medium were measured by ELISA. IL-17^+^ and IFN-γ^+^ cell were determined by intracellular staining followed by FACS analysis 5 days post-stimulation.

Mouse APCs (CD11c^+^ splenic cells) were separated from oxATP-treated or untreated immunized B6 mice 13 days post immunization using MACS column and cultured in the presence of LPS (100 ng/ml) for 48 h. Cytokine (IL-1, IL-6, L-12 and IL-23) levels in the culture medium were measured by ELISA.

### Assessment of the Effects of oxATP on Foxp3^+^ T Cells

CD3^+^ T cells were isolated from IRBP_1-20_ immunized B6 mice, with or without oxATP treatment, 13 days post-immunization. The percentage of Foxp3^+^ T cells among αβ T cells was determined by intracellular staining with PE-conjugated anti-Foxp3 Abs and APC-conjugated anti-mouse αβTCR Abs. To test the in vitro effect of oxATP, CD3^+^ T cells were cultured in a 24-well plate in the presence or absence of oxATP (80 μM). Five days later, the percentage of Foxp3^+^ among the responder T cells was determined by intracellular staining.

To test the inhibitory effect, αβ T cells and Foxp3^+^ T cells were co-cultured under Th1- or Th17-polarizing conditions. After 48 h, IFN-γ and IL-17 in the culture medium were measured by ELISA. The percentage of IFN-γ^+^ and IL-17^+^ T cells among the responder T cells was determined by intracellular staining 5 days after stimulation.

### Cytoplasmic Staining and Immunofluorescence Flow Cytometry

In vivo primed T cells were incubated for 5 days as described above, then activated T cells were separated using Ficoll gradient centrifugation and stimulated with 50 ng/ml of phorbol myristic acetate, 1 μg/ml of ionomycin, and 1 μg/ml of brefeldin A (all from Sigma) for 5 h in vitro. The cells were fixed and permeabilized overnight with Cytofix/Cytoperm buffer (eBioscience), and intracellularly stained with antibodies against IFN-γ, IL-17, or Foxp3. Data collection and analysis were performed on a FACS_calibur_ flow cytometer using CellQuest software.

### Statistical Analysis

All experiments were repeated 3 times. Experimental groups consisted of six mice. All measured data are expressed as the mean ± the standard deviation and analyzed by SPSS 17.0. ANOVA was used for multi-group comparisons. Each result was compared using the Student-Newman-Keuls test. A *P* value of <0.05 was considered significant.

## Results

### Treatment of B6 Mouse with an ATP Receptor Competitor—Oxidized ATP (oxATP) Almost Completely Abolished the Induced EAU in B6 Mouse

**Fig 1:**
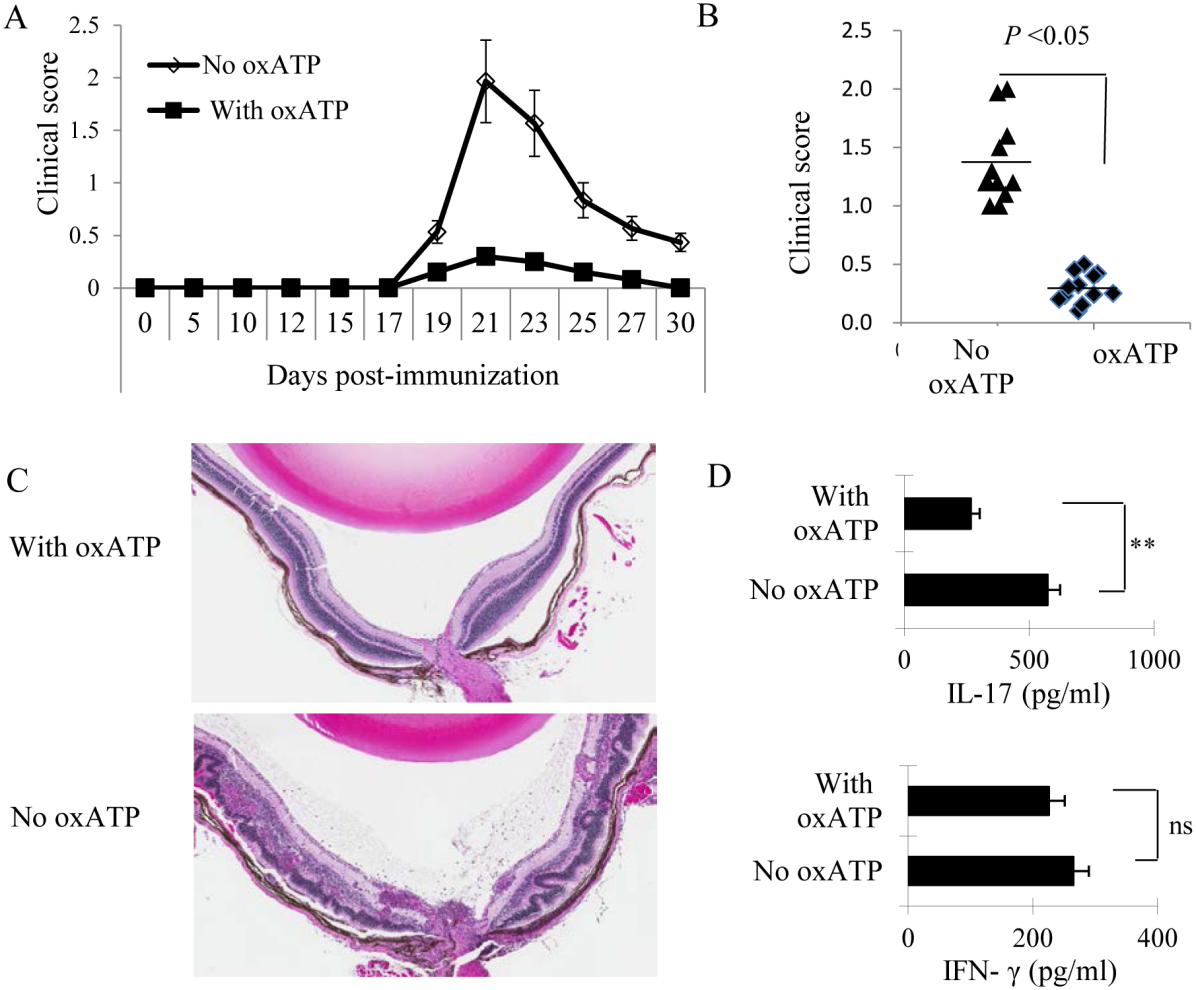
Injection of oxATP into EAU-prone B6 mice abolished EAU induction. A&B) Two groups of B6 mice (n = 6) were immunized with IRBP1-20/CFA and injected i.p. On day1 post-immunization, one group was injected i.p. with oxATP (300 μg/mice; twice a week), and the control group was injected i.p. with PBS only. EAU was clinically scored by fundoscopy. Results of three separate experiments were pooled for a statistical analysis (1B). C) On day 21 post-immunization, sample mice from each group were sacrificed and the eyes were subjected to pathological examination. H&E staining of eye sections from oxATP-treated and oxATP-untreated mice are shown. D) Serum cytokine (IFN-γ and IL-17) levels were measured by ELISA on day 13 post immunization. Blood samples collected from each mouse of the same experimental group were pooled and tested in triplicates by ELISA. The standard errors were calculated from triplicated samples. Data are from a single experiment, representative of three independent experiments. **P < 0.01, ns, not significant.

B6 mice were immunized with the uveitogenic peptide IRBP_1-20_ in CFA, then randomly divided into two groups (n = 6), one of which received injections of oxATP (300 μg/mouse, every three days) via i.p, starting 1 day post-immunization. The control group was treated with PBS. EAU was monitored by a combination of fundoscopy and pathologic examination. From each experimental group (n = 6), two mice were subjected to pathological examination 3 weeks after immunization, when EAU expression is maximized. The results showed that mice that received oxATP treatment had almost undetected EAU, as shown by fundoscopic (Fig 1A and 1B) and pathologic examination, whereas in the untreated mice massive inflammatory cell infiltration was found in the posterior chamber, mainly in the vitreous and retinal layers with serous exudates and subretinal bleeding (Fig 1C). Detection of serum cytokines showed that serum IL-17 was significantly decreased in oxATP-treated mice as compared to controls (Fig 1D).

### oxATP Preferentially Inhibited Th17 Autoimmune Responses

**Fig 2:**
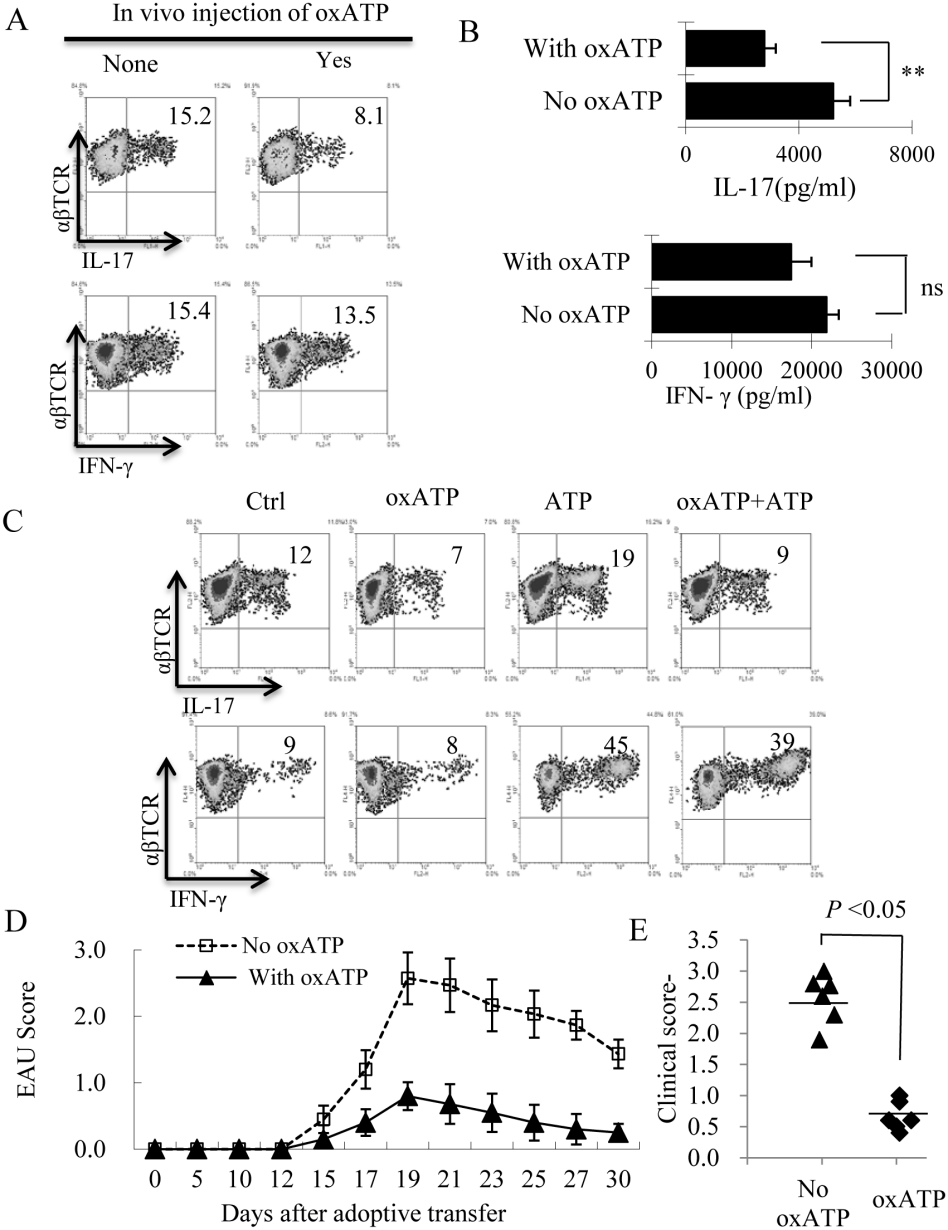
Administration of oxATP mitigated the autoreactive T cell response in the recipient B6 mice. A) Splenic T cells were isolated 13 days post-immunization from immunized B6 mice (n = 6) with (right panels) or without (left panels) oxATP administration. Cells obtained from all six mice in the same group were pooled, before the T cells are further enriched, stimulated with Ag/APCs, and subjected for phenotypic and functional analysis. The percentage of IL-17^+^ and IFN-γ^+^ cells among the proliferating T cells was assessed after 5-day in vitro stimulation under Th1 (lower panel) and Th17 (Upper panel) polarizing conditions by intracellular staining with PE-conjugated anti-αβTCR Abs and FITC-conjugated anti-IL-17 Abs (upper panels) or APC-conjugated anti-αβTCR and PE-conjugated anti-IFN-γ Abs (lower panels), followed by FACS analysis. B) IL-17and IFN-γ levels in the supernatant of in vitro cultured T cells after exposure to immunizing Ag and APCs for 48 h. **P < 0.01, ns, not significant. C) oxATP neutralized the enhancing effect of ATP on Th17, but not Th1, response. The responder T cells were prepared from immunized B6 mice, which were stimulated in vitro with the immunizing peptide and APCs, under polarizing T activation conditions, as described in Fig 2A, with or without a prior exposure to ATP (100 μM) and/or oxATP (80 μM). The proliferating T cells were assessed after 5-day in vitro stimulation by intracellular staining. D&E) IRBP-specific T cells isolated from oxATP-treated mice were poorly uveitogenic. After 2-day in vitro stimulation of αβ T cells with the immunizing peptides and APCs, 2 x10^6^ cells were adoptively transferred to naïve B6 via i.p. injection and EAU was scored by fundoscopy as described in Fig 1. Data are from one single experiment, representative of three independent experiments.

To determine the mechanism by which oxATP administration prohibited the induced EAU, we compared the Th1 and Th17 responses in oxATP-treated and untreated mice. At day 13 post-immunization (the time at which the highest T cell response is seen), responder T cells were purified from the spleen and draining lymph nodes of the immunized mice and stimulated in vitro with the immunizing peptide and APCs (irradiated spleen cells) under culture conditions that favor Th17 or Th1 autoreactive T cell expansion. The T cells separated by Ficoll gradient centrifugation were intracellularly stained with FITC-labeled anti-IFN-γ or anti–IL-17 antibodies and then examined for Th1 and Th17 responses. We found that the number of IL-17^+^ cells significantly decreased among the in vivo primed responder T cells of oxATP-treated mice (15.2% compared to 8.1% in controls; Fig 2A, upper panels); the IFN-γ^+^ cells, however, were only minimally affected (Fig 2A, lower panels). The cytokine production measured at 48 h after in vitro stimulation agreed with results obtained by intracellular staining. As shown in Fig 2B, responder T cells from oxATP-treated mice produced significantly less IL-17 than T cells from non-treated mice; whereas the IFN-γ production of responder T cells did not differ significantly between the two set. To determine whether the oxATP effect is mediated via ATP, we also tested whether the in vitro Th1/Th17 response in the absence or presence of ATP, oxATP and a combination of ATP and oxATP (Fig 2C). Our results showed that ATP enhanced both Th1 and Th17 response; the enhancing effect on Th17, but not Th1, response is neutralized by the oxATP. We have also compared the pathogenic activity of the IRBP-specific T cells isolated from treated and untreated mice. The T cells were obtained from spleens and draining lymph nodes. After 48 h stimulation with the immunizing antigen and APCs in vitro, 2 x 10^6^/recipient mouse separated T cells were adoptively transferred into naive B6 mouse, and the severity of induced EAU was evaluated. The results showed that IRBP-specific T cells isolated from oxATP-treated mice had significantly decreased ability to induce EAU upon adoptive transfer to naïve mice (Fig 2D and 2E).

### Effect of oxATP on DCs

**Fig 3:**
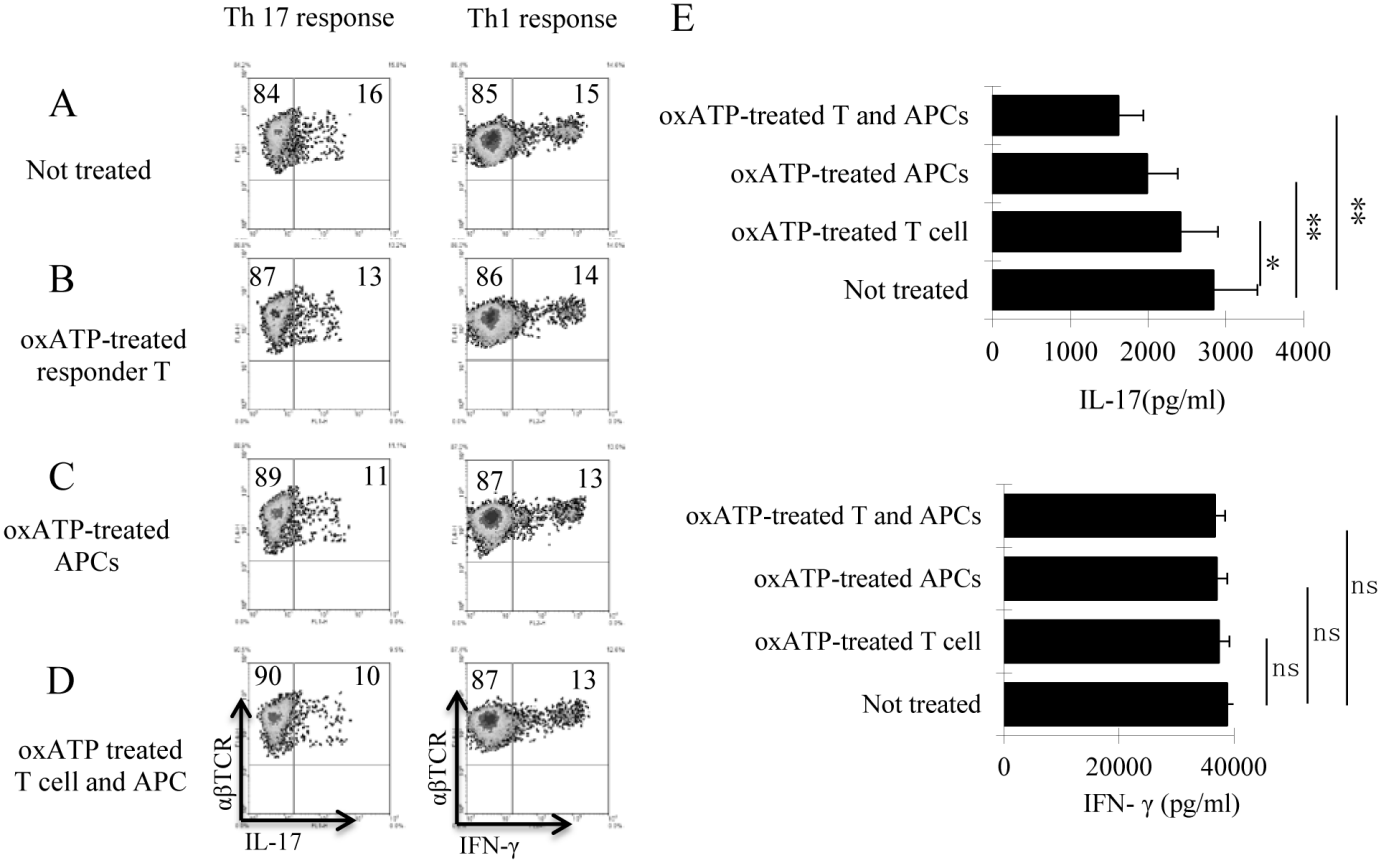
oxATP inhibited autoreactive T cell responses via both a direct effect on T cells and inhibiting DC function. A-D) Responder T cells and splenic APCs were isolated from immunized B6 mice, 13 days post immunization. Before co-incubation, T cells and DCs were either treated by oxATP (80 μM) or remained untreated. The stimulatory effect on T cells by the immunizing peptide and APCs were examined by co-culture of untreated responder T cells and APCs (3A); responder T cells treated but APCs untreated (3B), responder T cells untreated but APCs treated (3C), or both responder T cells and APCs were oxATP-treated (3D). After a 5-day co-incubation, the activated T cells were separated by Ficoll gradient centrifugation and staining for IL-17^+^ (among Th17 polarized stimulation, left panels of A-D) and IFN-γ^+^ cells (among Th1 polarized stimulation, right panels of A-D). E) IL-17and IFN-γ levels in the supernatant of cultured T cells after exposure to immunizing Ag and APCs for 48 h, were detected by ELISA in triplicates. * *P* < 0.05, **P < 0.01, ns, not significant.

To determine whether oxATP suppressed the autoimmune response by its effect on antigen-presenting DCs or by directly inhibiting autoreactive T cells, we examined the oxATP effect on T cell responses. The responder T cells were separated from immunized B6 mice 13 days post immunization. The cells were stimulated in vitro with the immunizing antigen and APCs (Fig 3A). To determine whether the inhibitory effect of oATP was mediated by its effect on APCs or responder T cells, the responder T cells and APCs were pre-treated by exposure to oxATP (80 μM), before the T cells were co-incubated with the APCs. After 5-day stimulation, the T cells were separated for intracellular staining with anti-IFN-γ or anti-IL-17 antibodies. The results show that pre-exposing either the responder T cells (Fig 3B) or APCs (Fig 3C) to oxATP significantly decreased Th17 responses; but when both APCs and T cells were treated (Fig 3D) with the testing dose of oxATP, the inhibition was significantly greater, indicating that oxATP has an inhibitory effect on both T cells and DCs. Cytokine production measured at 48 h after in vitro stimulation agreed with results obtained by intracellular staining (Fig 3E). Since oxATP treatment did not significantly affect the Th1 responses, inhibition caused by a toxic effect has been excluded.

**Fig 4:**
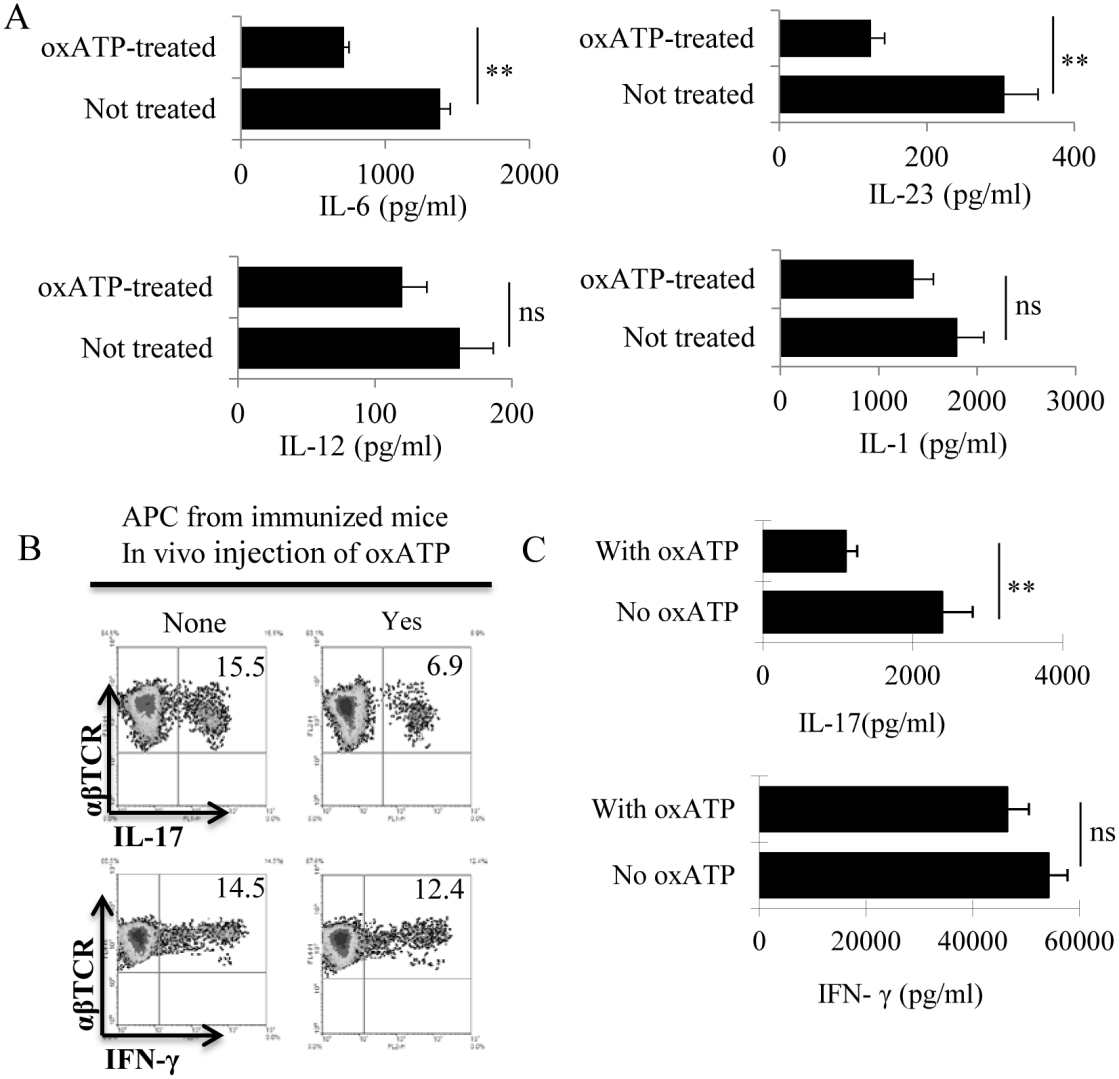
Splenic DCs of the oxATP recipients have decreased ability to produce IL-23 and IL-6 and were poor stimulators for Th17 autoreactive T cells. A) Splenic CD11c^+^ DCs were isolated from oxATP-treated (300 μg/mouse) and untreated immunized B6 mice 13 days post immunization, using MACS column. They were exposed to LPS (100 ng/ml) for 48h. Cytokines in the cultured cell supernatants were examined by ELISA. **P < 0.01, ns, not significant. B) In a 24-well plate, splenic APCs from oxATP-treated and untreated mice were compared for their effectiveness at stimulating Th1 and Th17 responses, by incubating with responder T cells isolated from immunized B6 mice, in the presence of the immunizing peptide (IRBP1-20). Five days after stimulation, the percentage of IL-17^+^ (under Th17 polarized conditions) and IFN-γ^+^ (under Th1 polarized conditions) cells among the responders was determined by intracellular staining followed by FACS analysis. C) IFN-γ and IL-17 amounts in the culture medium were measured by ELISA 48 h after in vitro stimulation. **P < 0.01, ns, not significant.

To determine the mechanism by which oxATP inhibited APCs’ antigen-presenting activity, we isolated splenic DCs from immunized mice with or without oxATP injections. Cytokine tests showed that the APCs obtained from oxATP-treated mice produced significantly smaller amounts of IL-23 and IL-6 as compared to the same cells derived from oxATP-untreated mice; however, the IL-1 and IL-12 producing ability of both APCs did not differ significantly (Fig 4A). The APCs from oxATP-treated mouse were poorly stimulatory for IL-17^+^ IRBP-specific T cells, as compared to the same cells obtained from oxATP-untreated mice (Fig 4B and 4C).

**Fig 5:**
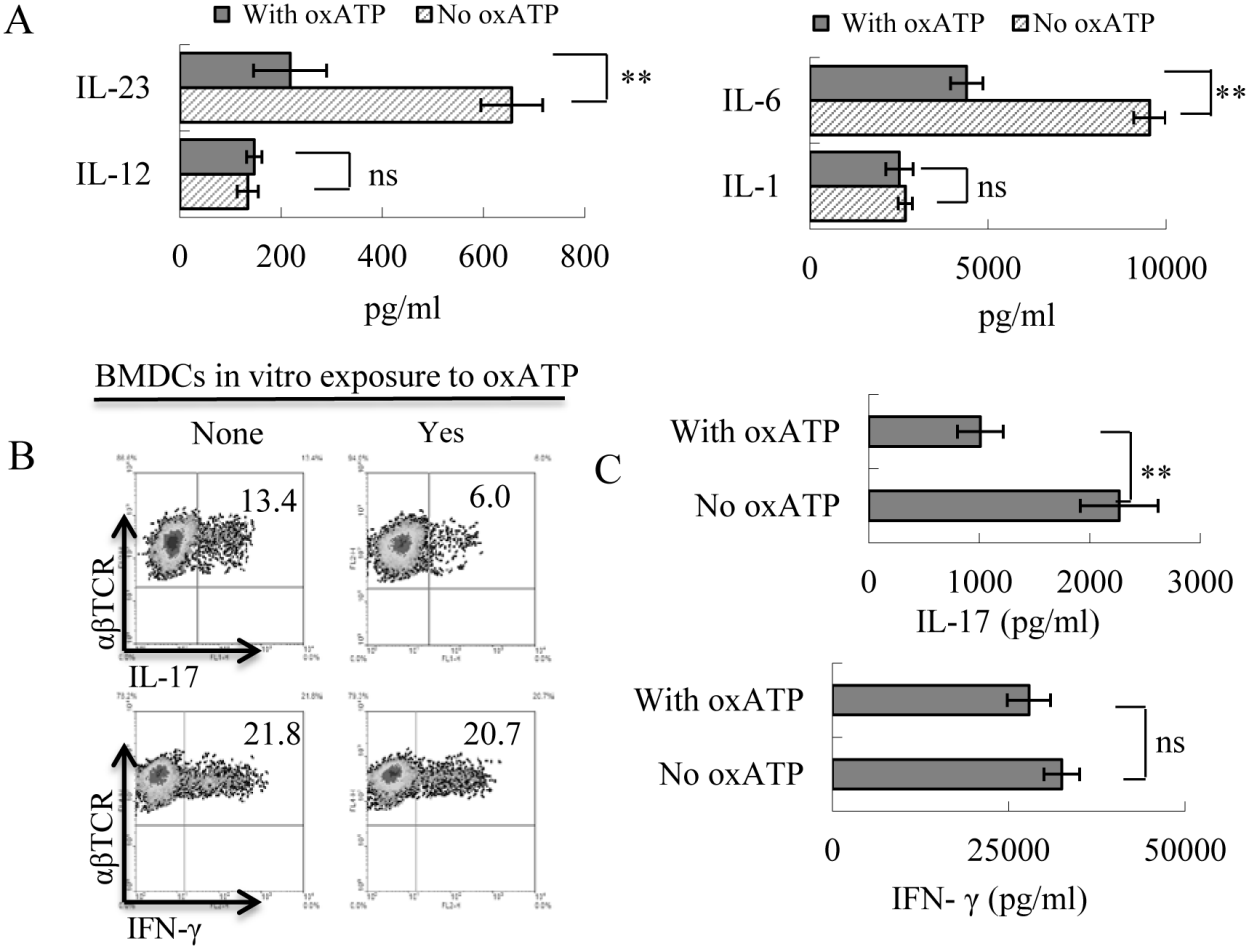
BMDCs produce decreased amounts of IL-23 and IL-6 and become poor stimulators for Th17 after exposure to oxATP in culture. Bone marrow cells from immunized B6 mice were cultured in GM-CSF and IL-4 (10 ng/ml)-containing medium for 5 days. IL-1, IL-6, IL-12 and IL-23 produced in the supernatant were assessed by ELISA, after 48 h exposure to oxATP (80uM). **P < 0.01; ns, not significant. B & C) Responder T cells were separated from immunized B6 mice 13 days post-immunization. T cells were co-cultured with BMDCs with or without a prior oxATP treatment, under Th17- or Th1-polarizing conditions. Five days after in vitro stimulation with the immunizing peptide and APCs, Th1 and Th17 responses were assessed by examining IL-17^+^ (Th17 polarized) and IFN-γ^+^ (Th1 polarized) T cells, followed by FACS analysis (5B). A 48 h cultured supernatant was assessed for cytokines by ELISA(5C). **P < 0.01; ns, not significant.

To further determine the effect of oxATP on DCs, we also examined the effect of oxATP on BMDCs prepared from a culture of bone marrow cells of immunized B6 mice. After a 5-day incubation in medium contain GM-CSF and IL-4 (10 ng/ml), in the absence or presence of oxATP (80 μM), the cytokine-producing ability was assessed after stimulation with LPS (100ng/ml). As shown in Fig 5A, the oxATP-treated BMDCs produced significantly smaller amounts of IL-23 and IL-6 as compared to the BMDCs cultured in the absence of oxATP. Functional tests showed that oxATP-treated BMDCs were poorly stimulatory for IL-17^+^ IRBP-specific T cells, as compared to untreated BMDCs, as shown by the percentage of IL-17^+^ cells among the responder T cells after intracellular staining (Fig 5B). Cytokine assessment of the responder T cell supernatants showed that the responder T cells produced significantly smaller amounts of IL-17 (Fig 5C) after stimulation by the oxATP-treated BMDCs, as compared to the same responder T cells stimulated by oxATP-untreated BMDCs. Likewise, the oxATP-treated BMDCs retained the ability to stimulate IFN-γ^+^ T cells.

### oxATP Is Inhibitory for Foxp3^+^ T Cells

**Fig 6:**
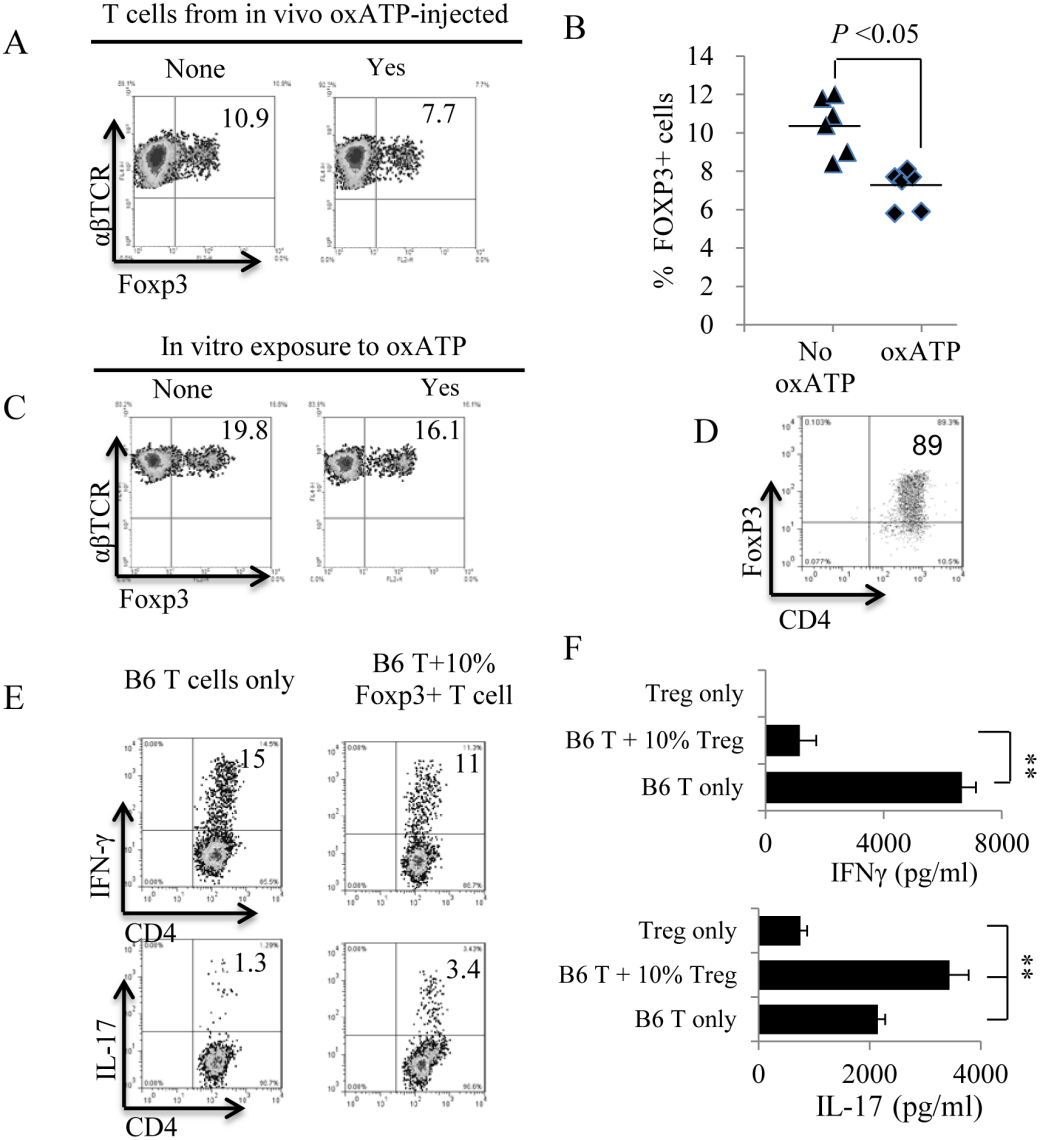
Inhibition of oxATP on Foxp3^+^ T cells. A & B) B6 mice were immunized with IRBP_1-20_/CFA with or without administration of oxATP (300 ug/mouse). Thirteen days post-immunization and immediately after separation of αβ T cells. Foxp3^+^ T cells were identified after a dual staining with anti-mouse αβTCR and anti-mouse-Foxp3 antibodies, followed by FACS analysis. C) oxATP has an inhibitory effect on Foxp3^+^ T cell. αβ T cells isolated from immunized mice were cultured for 5 days in the absence or presence of oxATP (80 μM) and proportional numbers of Foxp3^+^ T cells were compared among αβ T cells. D) Foxp3^+^ T cells were isolated from immunized B6 mice. CD3^+^ splenic T cells were cultured in vitro in medium containing very low dose (0.5 ng/ml) of IL-2. Five days later, 89% of the CD25^+^ cells separated using MACS column showed Foxp3^+^CD4^+^. E and F) Foxp3^+^ have a stronger inhibitory effect on Th1 response than on Th17 response. Responder T cells isolated from immunized B6 mice were stimulated with splenic APCs in the presence of the immunizing peptide, with or without adding 10% of Foxp3^+^ T cells. Five days after stimulation, IL-17^+^ (under Th17 polarized conditions) and IFN-γ^+^ (under Th1 polarized conditions) cells among the responders were determined by intracellular staining followed by FACS analysis (6E). IFN-γ and IL-17 amounts in the culture medium were measured by ELISA 48 h after in vitro stimulation (6F). **P < 0.01; ns, not significant.

To determine the possible mechanism of oxATP’s strong inhibitory effect on Th17 responses but not on Th1 responses, we investigated the oxATP effect on Foxp3^+^ regulatory cells. We first compared the number of Foxp3^+^ cells among the αβ T cells between oxATP-treated and untreated B6 mice. As demonstrated in Fig 6A and 6B, the αβ T cells of oxATP-treated mice contained significantly smaller numbers of Foxp3^+^ cells. We have also shown that the number of Foxp3^+^ cells decreased significantly if the responder CD3^+^ T cells were exposed oxATP (80 μM) (Fig 6C). Examination of the effect of Foxp3^+^ cells on Th1 and Th17 responses showed that the Foxp3^+^ cells are inhibitory for the Th1 response but have only a limited inhibitory effect on Th17 responses (Fig 6E and 6F).

## Discussion

Under pathologic conditions, a large amount of ATP is released into the extracellular compartment by injured and stressed cells [45,46]. The released eATP exerts multifaceted effects on various patho-physiological responses, including immune responses and inflammation [47,48]. Studies have shown that binding of eATP to P2 receptors modulated various inflammatory responses, including infections and tumors [1] and the inflammation induced during ischemia and reperfusion, as well as the inflammation that occurs in various disease states such as intestinal and lung diseases [49,50], type 1 diabetes, rheumatoid arthritis and multiple sclerosis [51] and graft-versus-host disease [28]. Among the P2 receptors, P2X7 receptor is primarily responsible for the proinflammatory effect of ATP [5,27,37,52].

To investigate whether manipulation of aberrant ATP signaling might be an effective therapy to control autoimmune disease, we evaluated the role of ATP and its antagonist on disease susceptibility of a well-established autoimmune EAU model. Our results showed that the administration of oxATP, a receptor antagonist specific for P2X7 ATP receptors, to EAU-prone B6 mouse consistently and almost completely abolished the induced EAU. We then examined the cellular and molecular mechanism by which oxATP administration abolished the induced EAU. The treated mice generated significantly fewer autoreactive T cells, but the decrease was mostly in the Th17 autoreactive T cells. Our results agree with previous reports that in vivo administration of oxATP blocked the onset of diabetes [3] and support the conclusion that eATP is essentially proinflammatory [3,6,13] while oxATP is anti-inflammatory. Mechanistic studies showed that the therapeutic effects involve a functional change in a number of immune cells, including DCs, T cells, and regulatory T cells. Not only can OxATP directly inhibit T cell response, but it also suppresses T cell activation by altering the APC function of DCs. Indeed, oxATP does not just block the binding of eATP to its receptor; it also inhibits the extracellular release of ATP. Conceivably, the binding of eATP to its receptor and the release of ATP are two events that might escalate each other’s activity [13].

Parallel tests of the effect on Th1 and Th17 autoimmune responses showed that Th1 responses were less affected by the treatment regimen than were the Th17 responses, a finding that appears to agree with the observation that DCs of oxATP-treated mice have greatly decreased IL-23 and IL-6 production; but their ability to produce IL-12 was only minimally affected. Additional study on the oxATP effect on Foxp3^+^ T cells showed that oxATP-treated mice have significantly lower Foxp3^+^ T cell activity; and the Foxp3^+^ T cells were inhibitory to Th1 responses, but not to Th17 responses. Conceivably, a diminished Foxp3^+^ T cell function offsets the inhibitory effect of oxATP on Th1, but not on Th17, response, because Foxp3^+^ cells are not significantly inhibitory for Th17 response in EAU; as a result, the net inhibitory effect of oxATP is stronger on Th17 than on Th1 response. Indeed, the finding that ATP enhanced the activation of Th17 T cells has been previously reported when it was demonstrated that the number of Th17 cells in germ-free mice increased after treatment with ATP but decreased after treatment with apyrase, which degrades ATP [6] and that Foxp3^+^ cells are less suppressive to Th17 than to Th1 response has been repeatedly observed by many groups [53–59]. In this study we showed that oxATP effectively neutralizes the enhancing effect of ATP on Th17 autoimmune response in an animal model of EAU. OxATP treatment should be considered as a means of control excessive Th17 immune responses in autoimmune diseases.

## Supporting Information

S1 File The ARRIVE guidelines checklist. (PDF)
